# Reduced procedural motor learning in deaf individuals

**DOI:** 10.3389/fnhum.2014.00343

**Published:** 2014-05-22

**Authors:** Justine Lévesque, Hugo Théoret, François Champoux

**Affiliations:** ^1^Centre de Recherche en Neuropsychologie et CognitionMontréal, QC, Canada; ^2^Centre de Recherche Interdisciplinaire en Réadaptation du Montréal Métropolitain, Institut Raymond-DewarMontréal, QC, Canada; ^3^École d’Orthophonie et d’Audiologie, Faculté de Médecine, Université de MontréalMontréal, QC, Canada

**Keywords:** deafness, cochlear implant, hearing loss, motor learning, plasticity, sensory deprivation, serial reaction time task

## Abstract

Studies in the deaf suggest that cross-modal neuroplastic changes may vary across modalities. Only a handful of studies have examined motor capacities in the profoundly deaf. These studies suggest the presence of deficits in manual dexterity and delays in movement production. As of yet, the ability to learn complex sequential motor patterns has not been explored in deaf populations. The aim of the present study was to investigate the procedural learning skills of deaf adults. A serial reaction-time task (SRTT) was performed by 18 deaf subjects and 18 matched controls to investigate possible motor alteration subsequent to auditory deprivation. Deaf participants had various degrees of hearing loss. Half of the experimental group were early deaf adults mostly using hearing aids, the remaining half were late-deaf adults using a cochlear implant (CI). Participants carried out a repeating 12-item sequence of key presses along with random blocks containing no repeating sequence. Non-specific and sequence-specific learning was analyzed in relation to individual features related to the hearing loss. The results revealed significant differences between groups in sequence-specific learning, with deaf subjects being less efficient than controls in acquiring sequence-specific knowledge. We interpret the results in light of cross-modal plasticity and the auditory scaffolding hypothesis.

## INTRODUCTION

Studies in the deaf suggest that cross-modal neuroplastic changes may vary across modalities (Bavelier and Neville, 2002). Sensory and motor outcomes seem to lack uniformity, as they vary between the heightening and lowering of abilities (for a review, see Collignon et al., 2011). In the visual domain, deaf individuals manifest both better and worse visual skills than hearing controls. Bavelier et al. (2006) have proposed that changes in visual cognition are selective to those attentionally demanding aspects of vision that would normally benefit from auditory-visual convergence. In the motor domain, Savelsbergh et al. (1991) have suggested that the lack of early auditory input could contribute to the motor delays in basic motor tasks observed in deaf children. Research has since shown that deaf children perform on average significantly worse than hearing children on different standardized tests of motor development (Dummer et al., 1996; Gheysen et al., 2008). More specifically, several studies of motor capacities in deaf children have reported deficits in general dynamic coordination, visual-motor skills, balance, ball catching abilities, as well as slower reaction times and speed of movement execution (Wiegersma and Van der Velde, 1983; Savelsbergh et al., 1991; Siegel et al., 1991; Hartman et al., 2011). In deaf adults on the other hand, motor capacities have not been extensively explored, but some studies suggest normal visual-motor skills in deaf individuals who are native signers (Hauser et al., 2007). Higher-level motor processing has also been examined in deaf children. Schlumberger et al. (2004) reported that deaf children showed delays in the development of complex movement production and Conway et al. (2011a) revealed sequencing disturbances in deaf children’s performance at a finger tapping task. However, as of yet, the ability to learn complex motor sequential patterns has not been explored in deaf populations.

Several studies suggest that profound deafness may result in disturbances in non-auditory abilities related to serial order information (Knutson et al., 1991; Pisoni and Cleary, 2004; Horn et al., 2005), although there is some debate as to whether auditory deprivation is the basis of such disturbances (Thorpe et al., 2002; Lyness et al., 2013; Dye and Hauser, 2014). In particular, Conway et al. (2011b) have reported deficits of visual implicit learning abilities in deaf children on a color-sequence task. They have proposed that exposure to sound, a temporally arrayed signal, provides important experience with learning of sequential patterns in the environment (a sort of “auditory scaffolding”) and that a lack of experience with sound at a young age may delay the development of domain-general processing skills of sequential patterns including non-auditory abilities (Conway et al., 2009). Considering the findings of motor deficits along with disturbances in non-auditory complex sequencing in deaf children, sequential motor learning skills may also be affected by deafness. Moreover, higher-level motor processing skills have only been tested in deaf children. The adult deaf population offers the opportunity to investigate whether motor learning deficits are also present at an adult age and if other factors, such as age of onset of the hearing loss, the duration of auditory deprivation period or the use of compensatory technologies to restore auditory function (i.e., use of a hearing-aid or a CI) modulate motor skill acquisition during development.

The aim of this study was to investigate the procedural learning skills of deaf adults. Motor capacities in profoundly deaf adults was assessed with the serial reaction time task (SRTT: Nissen and Bullemer, 1987). The SRTT, a choice reaction time task, requires participants to indicate the position of a visual stimulus through a motor action. Visual stimuli appear at one of several spatial locations and participants are required to indicate this location through motor movement as quickly as possible. The experimental design contains a sequence of visual stimuli, which is repeated. Participants are naïve to this experimental parameter. The SRTT is believed to involve two types of learning: sequence-specific learning and non-specific learning. The former is measured through reaction time improvement following the repetition of the visual-motor sequence. The latter is considered to be a general decrease in reaction time throughout the task. Beyond examining if the general delays in motor tasks observed in deaf individuals are related to procedural learning, the many features of hearing loss that might be responsible for increased or decreased motor learning (i.e., the duration of auditory deprivation, the nature, degree and age of onset of sensory loss and the duration of profound deafness without using hearing devices) were also investigated.

## MATERIALS AND METHODS

### PARTICIPANTS

Eighteen deaf individuals with various degrees of hearing loss (6 males and 12 females) aged 20–65 (mean age = 36) were recruited for this study. An equal number of normal-hearing control participants matched for age and sex served as controls. All participants were right-handed. All deaf participants had a severe to profound (auditory detection threshold beyond 71 dB HL) bilateral sensorineural hearing loss. Nine participants had a CI. No participant had two CIs. For all deaf individuals using a CI and control subjects, pure-tone detection thresholds were within normal limits (30 dB HL or less) at octave frequencies ranging from 250 to 6000 Hz. As for the other deaf individuals, all except three individuals (D1, D3, and D7) were routinely using hearing devices. Auditory performance with hearing devices appeared to be greatly variable, from being only able to detect loud sounds, to being able to identify monosyllabic words without visual cues. **Table 1** presents the clinical profile of the deaf participants. All participants gave their written informed consent and the ethics committee of the Faculty of Medicine of the University of Montreal approved the protocol.

**Table 1:** Clinical profile of the deaf participants.

Subjects	Sex	Age	Age at onset of deafness (years)	Cause of deafness	Deafness duration (years)	Duration of deafness without using hearing devices (years)	Principal mode of communication	Duration of CI use
D1	F	25	0 (Congenital)	Hereditary	25	25	Manual	–
D2	M	32	0 (Congenital)	Hereditary	32	0	Manual	–
D3	M	20	0 (Congenital)	Hereditary	20	20	Manual	–
D4	F	30	0 (Congenital)	Hereditary	30	3	Oral	–
D5	F	45	0–22 (Progressive)	Hereditary	45	0	Oral	–
D6	F	33	0 (Congenital)	Ototoxicity	33	3	Oral	–
D7	F	47	0 (Congenital)	Anoxia	47	7	Oral	–
D8	F	26	0 (Congenital)	Ototoxicity	26	0	Oral	–
D9	F	28	0 (Congenital)	Unknown	6	0	Oral	–
CI0	F	34	14–29 (Progressive)	Hereditary	20	3	Oral	5
CI1	F	64	19–55 (Progressive)	Hereditary	45	1	Oral	9
CI2	F	25	9–16 (Progressive)	Hereditary	16	1	Oral	9
CI3	M	53	39–44 (Progressive)	Unknown	14	0	Oral	9
CI4	F	38	7–31 (Progressive)	Unknown	31	1	Oral	7
CI5	F	36	21–27 (Progressive)	Unknown	15	1	Oral	9
CI6	M	23	5–18 (Progressive)	Hereditary	18	1	Oral	5
CI7	M	66	22–58 (Progressive)	Meningitis	44	0	Oral	8
CI8	M	25	9–16 (Progressive)	Hereditary	16	1	Oral	9

*D = deaf individual without cochlear implant; CI = cochlear implant user.*

### PROCEDURE AND STIMULI

The SRTT was presented on a computer with SuperLab software (version 4.5.3; Cedrus, San Pedro, CA, USA). The task consisted of the visual presentation of four horizontally aligned dots. Each dot represented a number from 1 to 4 on the computer’s keyboard. The left-most dot was associated with “1,” the second left-most dot was associated with “2,” the second right-most dot was associated with “3,” and the right-most dot was associated with “4” on the computer keyboard. The visual stimuli, in this case asterisks, varied from the four possible positions. The apparition of an asterisk in one of the four positions indicated which key to press. Participants were told to press the key on the keyboard corresponding to the position of the asterisk as fast as possible with the appropriate finger (for the right hand: index at position 1, middle finger at position 2, ring finger at position 3, and little finger at position 4). The asterisk changed position only when the correct key was pressed. When the participant made a mistake, the stimulus stayed at the same position until the participant gave the correct answer.

The task consisted of 14 blocks to be completed with the right hand. For each block, twelve finger positions designated by asterisks were presented (ex: ·*·· to represent a key press by the middle finger). Each block consecutively displayed 12 such sequences. Some blocks presented a random sequence of key-press stimuli, whereas others followed a predetermined sequence corresponding to the following positions: 4–2–3–1–1–3–2–1–3–4–2–4. The task included an initial practice block of random-order key presses (R1) to familiarize participants with the task. A second block of random-order key presses (R2) was used to determine initial performance. Following these two initial blocks, 10 trials of the predetermined sequence order (A1–A10) were presented with a random block (R3) inserted between A5 and A6. One last block of random-order key presses (R4) followed A1–A10 and R3. The reaction time, defined as the mean time taken by participants to press the correct key, was measured for each block.

### DATA ANALYSIS

The learning effect was calculated as the difference in reaction time between the first (A1) and the last block (A10) of the repeated pre-determined sequence blocks. Non-specific learning was calculated as the difference in reaction time between the second (R2) and last random blocks (R4). To eliminate any learning effect that may arise due to habituation during task execution and to measure sequence-specific learning, the difference in reaction times between the last random block (R4) and the sequential block that preceded it (A10) was calculated. An analysis of variance based on groups, block variables, and the sequence type (repeated vs. random) was performed. To examine if control and deaf individuals had similar improvements following training, we examined the percent change in median reaction times between the first and last training blocks [(A1-A10)/A1]. Percent change in median reaction times in the last block of the repeating sequence relative to the subsequent random block [(A10-R4)/A10] was also calculated.

Mean number of errors (wrong key presses) was also calculated to ensure stable overall accuracy during the course of the experiment. Finally, the performance level of deaf individuals was examined in relation with the characteristics of the hearing loss.

## RESULTS

### REACTION TIME IN SEQUENCE AND RANDOM BLOCKS

**FIGURE 1:**
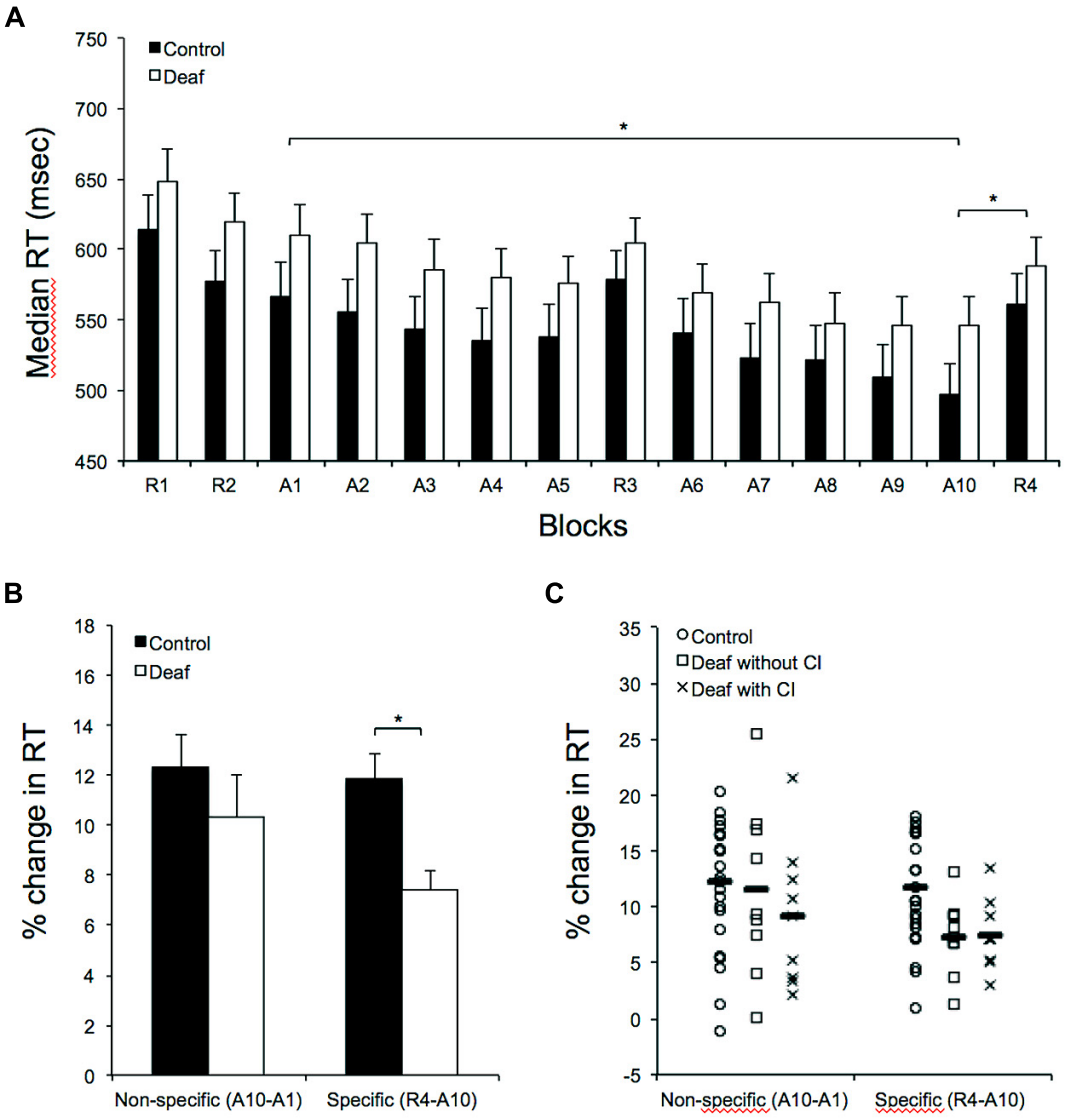
**Results on the serial reaction time task in controls (*n* = 18) and in deaf individuals (*n* = 18). (A)** Response time (RT) in random and sequence blocks during the SRTT. The abscissa shows block type in temporal order, and the ordinate shows median RT. Note the significant, progressive RT shortening in sequence A (A1–A10 blocks) as well as the significant sequence-specific learning when controlling for task exposure (A10–R4) in both groups. **(B)** This panel depicts similar RT reductions (in percent change) in control relative to the deaf after 10 training blocks (A1 vs. A10). The histogram, however, illustrates the significantly greater sequence-specific RT increase (in percent change) from the last training block (A10) to the immediately following random block (R4) in control relative to the deaf Individuals. **(C)** Individual results of the control and the deaf with or without a cochlear implant (CI). Group averages are shown with horizontal bars. (*) Corresponds to *P* < 0.001.

Performance at the SRTT is shown in **Figure 1**. As expected, both groups showed progressive decreases in reaction time between the first (A1) and the last training block (A10; **Figure 1A**). Both groups showed explicit non-specific learning (R2–R4) and showed sequence-specific learning when controlling for task exposure (A10–R4; **Figure 1A**). Analysis confirmed that both groups showed some level of motor learning during the task. Significant differences in reaction times were found for each group when comparing blocks A1–A10 (Control: *t*_17_ = 8,536; *P* < 0.001; Deaf: *t*_17_ = 5,853; *P* < 0.001), R2–R4 (Control: *t*_17_ = 3,059; *P* = 0.007; Deaf: *t*_17_ = 2,947; *P* = 0.009), and R4–A10 (Control: *t*_17_ = 13,802; *P* < 0.001; Deaf: *t*_17_ = 10,213; *P* < 0.001).

Further analyses were conducted to explore possible group differences. An analysis of variance with group (control; deaf) as between subjects factor and training block (A1–10) as a within-subjects factor was conducted. The main effect of group on median reaction times was not significant (*F*_1,__34_ = 1.722; *P* = 0.198; $\eta_{p}^{2}$ = 0.048). As expected, there was a main effect for training block (*F*_1,__34_ = 36.114, *P* < 0.001, $\eta_{p}^{2}$ = 0.515) and the interaction between factors was not significant (*F*_1,__34_ = 1.127, *P* = 00.343, $\eta_{p}^{2}$ = 0.032). An analysis of variance with group (control; deaf) as between-subjects factor and random block (R1, R2, R3, and R4) as a within-subjects factor was also conducted. Again, the main effect of group on median reaction times was not significant (*F*_1,34_ = 1.272; *P* = 0.267; $\eta_{p}^{2}$ = 0.036). There was a main effect for training block (*F*_1,__34_ = 21.018, *P* < 0.001, $\eta_{p}^{2}$ = 0.382) and the interaction between factors was not significant (*F*_1,__34_ = 0.556, *P* = 0.645, $\eta_{p}^{2}$ = 0.016). There was no group difference in mean response accuracy during the task (*F*_1,3__4_ = 0.128; *P* = 0.723).

### PERCENT CHANGE IN REACTION TIME: NON-SPECIFIC AND SPECIFIC EFFECT OF LEARNING

In order to determine which of the conditions was different between groups, we then performed two separate ANOVAs. The effect of the training block (i.e., non-specific effect of learning) was not significant when percent change in reaction times [(A1-A10)/A1] for each participant was used to compute a between-group (control; deaf) analysis of variance (*F*_1,3__4_ = 0.894; *P* = 0.351; **Figure 1B**). This indicates that control and deaf individuals improved similarly after 10 training blocks. In opposition, the results suggest that deaf individuals benefited significantly less than controls from 10 training blocks of a repeating sequence relative to the subsequent random block (i.e., specific effect of learning). Indeed, the sequence-specific effect of learning was significant when percent change in reaction times [(A10-R4)/A10] for each participant was used to compute the analysis of variance (*F*_1,3__4_ = 12.682; *P* = 0.001; **Figure 1B**).

The performance level of deaf individuals was examined further in relation with CI use. The non-specific effect of learning (*F*_1,__16_ = 0.515; *P* = 0.484) and the sequence-specific effect of learning (*F*_1,__16_ = 0.014; *P* = 0.907) were not significant when percent change in reaction times for each participant was used to compute between-group (CI users; non-users) analysis of variance (**Figure 1C**). Finally, we also examined the results in relation to the characteristics of the hearing loss. After correcting for multiple comparisons (corrected *p*-value = 0.0125), there were no significant relationships between the sequence-specific effect of learning and the characteristic of the hearing loss, including the duration of deafness, the age at onset of hearing loss or the duration of profound hearing loss for individuals not using a hearing device (i.e., hearing aid or CI). There was also no significant relationship between the sequence-specific effect of learning and the duration of CI use.

## DISCUSSION

In the present study, the SRTT was used to investigate whether procedural motor learning differs in the deaf compared to hearing individuals. Although we recognize that the SRTT has both motor and perceptual learning components, we hold, in agreement with Willingham (1999) and Deroost and Soetens (2006), that the task concerns primarily motor learning. Research has shown perceptual learning to be rather limited and secondary to response-related learning in complex sequencing tasks (Kelly and Burton, 2001; Remillard, 2003; Deroost and Soetens, 2006). Moreover, a recent study by Hallgató et al. (2013) has provided evidence of differential consolidation of perceptual and motor learning at a modified SRTT, with motor knowledge transferring more effectively than perceptual knowledge with time. Their results further support the notion that motor learning plays a primary role in sequential learning. Motor learning was observed amongst participants of both groups in our study. A significant difference was measured for reaction times between the first (A1) and last blocks (A10) of the repeated sequence but also between the last repeated sequence block (A10) and the last random block (R4). The difference between A10 and R4 indicates sequence-specific learning on this motor task, while eliminating any learning effect due to habituation and knowledge of the task. A non-specific learning effect was measured by a decrease in reaction time between the second (R2) and last random blocks (R4). This reaction time difference between R2 and R4 was significant for both groups, which likely reflects an improvement of the skill involved in selecting and pressing the correct keys throughout the experimentation (Willingham, 1999). Yet, although no significant difference was found between deaf and hearing subjects for the training blocks, it should be mentioned that the reported effect sizes suggest some differences may be present, but obscured by variability. Beside this lack of evidence on baseline reaction time for both groups, a significant difference was observed when we compared the two groups in sequence-specific learning; participants in the deaf group were significantly less efficient than controls in acquiring sequence-specific knowledge. There were no correlations between task performance in the deaf group and other characteristics of the hearing loss.

Our results stress the importance of acquiring knowledge on the motor reorganization that occurs following a period of prolonged deafness. The current body of research studying how deafness alters the perception of the external world suggests that a prolonged period of deafness can lead to significant alterations in sensory processing (for a review, see Bavelier and Neville, 2002; Collignon et al., 2011). Until now, deafness rehabilitation methods have reflected these findings and have generally been met with success. Research data, particularly in the visual domain, have helped in predicting functional outcomes on an individual basis prior to cochlear implantation (e.g., Giraud and Lee, 2007; Lee et al., 2007; Buckley and Tobey, 2011; Sandmann et al., 2012). Research data have also allowed more effective patient counseling and expectation management. Moreover, these data have led to the understanding of why some individuals make better use of their CI following surgical implantation while others struggle in very specific perceptual situations (see Landry et al., 2012).

The results reported in this study are in agreement with what has been found in previous studies examining sequential learning in the deaf. According to Conway et al. (2011b), deaf individuals are less skilled at learning implicit sequences, but perform normally in explicit non-specific learning. These authors showed evidence of this phenomenon for visual implicit sequence learning. Importantly, visual sequential abilities correlated with duration of auditory exposure; greater auditory exposure led to improved skills for sequential processing. Conway et al. (2009) have proposed that exposure to sounds at a young age supports the learning and representation of sequential patterns and have termed the role of sound in the development of sequencing abilities the “auditory scaffolding” hypothesis. According to this assumption, auditory scaffolding is absent in individuals with congenital profound deafness. This absence would thus result in brain reorganization and disturbance to non-auditory sequencing abilities. The experimental group in this study and the study by Conway et al. (2011b) did not share the same characteristics of deafness, however. Participants in their study were children between the ages of 5 and 10 with a CI. The participants in the present study were all adults over 20 years of age with heterogeneous audiological profiles. Most importantly, only half of the participants were using a CI and seven of the nine remaining participants were fitted with hearing aids. Still, the procedural learning difficulty reported here in deaf adults using various hearing aids technology corroborates the auditory scaffolding hypothesis as derived using data from children with CIs.

The possible factors explaining performance in the deaf should be interpreted cautiously. In the present study, all deaf participants without a CI acquired deafness before the age of six and all deaf participants with a CI acquired deafness after the age of five (range 5–39 years old). However, all except two participants had more than 15 years of hearing loss and only two had more than seven years of profound deafness without using hearing devices. In that regard, CI users and non-users were identical. Relationship between learning performance and the use of a CI is also difficult to interpret. Indeed, all participants were implanted at least at 15 years of age and the duration of CI use was very similar across individuals (between 5 and 9 years). Due to the similar time frame of these two variables, distinguishing between the duration of CI use and the impact of the age at the moment of the cochlear implantation is also impossible to determine.

This study shows that deafness can lead to problems in sequence processing that may reflect deficits in motor learning. This contributes to the small but growing literature on sequence processing and sequence learning and the role of audition in shaping such processes. In order to support the implication of each features of the hearing loss in procedural learning, however, further experiments comprising of deaf individuals with various degrees of language exposure (see Lyness et al., 2013) and auditory exposure will be needed.

## Conflict of Interest Statement

The authors declare that the research was conducted in the absence of any commercial or financial relationships that could be construed as a potential conflict of interest.
